# Experimental conditions shape *in vitro* formation of murine platelet-leukocyte aggregates

**DOI:** 10.3389/fimmu.2025.1637038

**Published:** 2025-09-02

**Authors:** Silvia Maria Grazia Trivigno, Alice Assinger, Waltraud Cornelia Schrottmaier

**Affiliations:** ^1^ Institute of Vascular Biology and Thrombosis Research, Centre of Physiology and Pharmacology, Medical University of Vienna, Vienna, Austria; ^2^ University School for Advanced Studies IUSS and Department of Biology and Biotechnology, University of Pavia, Pavia, Italy

**Keywords:** platelet-leukocyte aggregate, blood draw, anticoagulant, delayed processing, temperature, method optimization, murine model

## Abstract

**Background:**

Platelets interact with leukocytes to fine-tune their functions, thus providing essential regulation of (patho-) physiologic immune responses in various diseases. Circulating platelet-leukocyte aggregates (PLAs) represent a sensitive biomarker to estimate disease severity both in patients and murine models. However, a limited understanding of the sensitivity of PLA measurements to methodological variables may undermine their accuracy and comparability.

**Objectives:**

To elucidate how blood draw techniques, anticoagulation, processing delay and assay temperature affect murine platelet-leukocyte interactions.

**Methods:**

Murine blood was obtained via retro-orbital, *vena cava* or cardiac puncture, anticoagulated with heparin, citrate or acid-citrate-dextrose (ACD) +/- recalcification and stored for 30-120 min before stimulation at room temperature or 37°C with adenosine diphosphate (ADP), cross-linked collagen-related peptide (CRP-XL) and protease-activated receptor 4-activating peptide (PAR4-AP). PLA formation and leukocyte activation were analyzed by flow cytometry.

**Results:**

Basal PLAs were minimally affected by blood sampling and anticoagulant, though delayed processing significantly raised basal PLAs. Agonist-induced PLA formation was independent of anticoagulation, and sampling technique did not affect ADP- or PAR4-AP-induced PLA levels. However, CRP-XL sensitivity was elevated in blood obtained by cardiac puncture. Contrarily, both delayed processing and stimulation at 37°C impaired CRP-XL sensitivity, but augmented ADP and PAR4-AP responses. Regulation of leukocyte activation did not follow PLA patterns, with monocytes and neutrophils displaying distinct susceptibility to anticoagulation, storage and temperature.

**Conclusions:**

Variations in preparing murine blood samples exert distinct influences on platelet-leukocyte interactions *in vitro*, underlining the critical need for fastidious assay optimization to support data reproducibility and comparability.

## 1 Introduction

Platelets are essential mediators in hemostasis, orchestrating (patho-) physiologic pro-thrombotic processes and preserving vascular integrity. However, platelets also retain immunomodulatory functions, inherited from their evolutionary ancestors, and thus represent a highly sensitive first line of defense against invading pathogens (1). Platelets regulate leukocyte responses via soluble mediators and by forming platelet-leukocyte aggregates (PLAs) (2). These hetero-aggregates are upregulated in a plethora of pathologic conditions, including cardiovascular and inflammatory diseases (1, 3), acute ischemic stroke (4), cancer (5), viral infections (6–8) and sepsis (9) and have garnered interest as therapeutic targets and biomarkers (10, 11).

The initial tethering of platelets to leukocytes is mediated by platelet P-selectin (CD62P) binding to leukocyte P-selectin glycoprotein ligand-1 (PSGL-1) with varying binding affinities leading to preferential formation of platelet-monocyte aggregates (PMAs) and platelet-neutrophil aggregates (PNAs) (12, 13). Further receptors that stabilize PLAs include CD11b/CD18 (Mac-1) on leukocytes and CD40 ligand (CD40L) and activated glycoprotein (GP) IIb/IIIa on activated platelets (14–16). Accordingly, PLA formation critically depends on the activation of platelets over leukocytes (17), enabling platelets to fine-tune pro-inflammatory leukocyte responses such as CD11b activation/upregulation, CD62L shedding, and monocyte polarization, a process termed thromboinflammation (3, 18–20).

Increasing recognition of the (patho-) physiologic impact of thromboinflammation has sparked an interest to investigate platelet-leukocyte interactions in patients and murine disease models alike. Most commonly, PLAs are quantified in blood via flow cytometry as it represents a quick, noninvasive and robust technique, though the lack of uniform laboratory protocols makes direct comparisons between studies challenging (21, 22). Platelets are highly sensitive to changes in their microenvironment and their functional responses are easily influenced by methodological differences, such as blood sampling technique, anticoagulation, pH, temperature and storage (23–30). While effects on pro-thrombotic platelet responses are well-documented, little is currently known about the influence of procedural details on their immunomodulatory functions. We could recently show that platelet count, pH and temperature impact platelet binding and platelet-mediated activation of neutrophils and monocytes in human blood (27). Platelet and leukocyte proteomes and/or transcriptomes are highly conserved and the main receptors facilitating PLA formation are consistent between mice and humans, but substantial differences persist e.g. regarding signaling enzymes, receptor expression and leukocyte subpopulation composition, which might affect sensitivity to external stimuli (21, 31–33). Most prominently, human and murine platelets differ in their expression of protease-activated receptors (PARs) and Fc receptors, with human platelets expressing PAR1 and PAR4 as well as FcαRI, FcγRIIA and FcϵRI, whereas murine platelets express PAR3 and PAR4 and no Fc receptors (34). However, they also vary in expression levels of e.g. purinergic receptors, protein kinase C and phospholipase C isoforms and cytoskeletal talin (33, 35) and in the structure of some membrane receptors, e.g., GPVI (36). In addition, PLA levels may be influenced by higher platelet counts and by differences in leukocyte composition with lymphocytes representing the dominant circulating subpopulation in mice (21). Hence, independent studies on how procedural details modulate platelet-leukocyte interactions in murine setting are required in order to improve experimental design and advance accuracy and comparability of data.

This study aimed to elucidate how common protocol variations in blood sampling, anticoagulation, processing time, and temperature modulate immunomodulatory functions of murine platelets *in vitro*. By evaluating platelet binding of neutrophils and monocytes, their expression of CD11b and CD62L and monocyte polarization into Ly6C+ subsets upon platelet stimulation, we found that effects on PLA formation were often uncoupled from changes in leukocyte activation and polarization.

## 2 Materials and methods

### 2.1 Animals

C57BL/6J mice were bred at Medical University of Vienna (Austria) under standard specific-pathogen-free conditions with access to food and water ad libitum and 12h/12h light/dark cycle. Animal experiments were approved by the Animal Care and Use Committee of the Medical University of Vienna and the Austrian Ministry of Sciences (BMBWF-2024-0.019.492) in accordance with the EU Directive 2010/63 for the protection of animals used for scientific purposes. Experiments were performed on male and female mice aged 12-30 weeks.

### 2.2 Anesthesia and blood draw

For retro-orbital blood (RO) draw, mice were anesthetized by inhalation of isoflurane (Forane; Baxter Healthcare Corporation). Blood (300 µl) was drawn using heparinized microhematocrit tubes (Brand) into plastic tubes and immediately mixed with 60 µl anticoagulant dilution. For drawing blood from the inferior *vena cava* (VC) or heart (H), mice were injected intraperitoneally with an overdose of ketamine (250µg/g; Ketasol; Livisto) and xylazine (25µg/g; Xylasol; AniMedica). 500 µl blood was drawn from the VC or H using a 27G needle into syringes containing 100 µl prepositioned anticoagulant within the dead volume. Mice were immediately euthanized by cervical dislocation after blood draw. Of note, as anesthesia may influence hemodynamics and coagulation, all mice were anesthetized with ketamine/xylazine when comparing sampling methods (37, 38). Blood samples were kept at room temperature (RT; 22-26°C) until analysis.

### 2.3 Anticoagulation

Per default, blood was anticoagulated with 25 U/ml heparin (Biochrom). To test effects of different anticoagulants, acid-citrate-dextrose (ACD; Sigma-Aldrich) or 3.2% sodium citrate (Greiner) were added immediately after blood draw in a ratio of 1:10 and 2 mM CaCl_2_ were supplemented as indicated in the figures and figure legends.

### 2.4 *In vitro* stimulation

Whole blood was stimulated for 15 min with phosphate-buffered saline (PBS) or adenosine diphosphate (ADP; Sigma-Aldrich), cross-linked collagen-related peptide (CRP-XL; Cambcol), or PAR4-activating peptide (PAR4-AP, AYPGKF-NH2; AnaSpec) at RT or 37°C as indicated in the figures and figure legends. Cells were incubated with labelled antibodies (20 min) and fixed in 1% formaldehyde (10 min). Erythrocytes were lysed using lysis buffer (150 mM NH_4_Cl, 10 mM KHCO_3_, 0.1 mM Na_2_EDTA, pH 7.4; 10 min) and non-lysed cells were pelleted (600×g, 5 min) and resuspended in PBS.

### 2.5 Flow cytometry and gating strategy

PLA formation and leukocyte activation were analyzed using the following antibodies (all BioLegend): anti-CD41-AF700 (1:100, MWReg30), anti-Ly6G-APC (1:200, 1A8), anti-CD115-BV421 (1:100, AFS98), anti-CD11b-PE-Cy7 (1:400, M1/70), anti-CD62L-FITC (1:100, MEL-14), and anti-Ly6C-BV605 (1:100, HK1.4). Samples were measured on a CytoFLEX S (V4-B2-Y4-R3) flow cytometer and analyzed with CytExpert 2.4 software (both Beckman Coulter).

Myeloid cells were identified as CD11b-positive singlet events and further discriminated into neutrophils and monocytes based on the expression of Ly6G and CD115, respectively. PNAs and PMAs were assessed as the percentage of neutrophils or monocytes positive for CD41 based on fluorescence-minus-one staining. Neutrophil and monocyte activation was further evaluated by assessing CD11b expression and CD62L shedding, quantified as mean fluorescence intensity (MFI). To analyze monocyte polarization, CD115-positive events were discriminated via Ly6C expression into classical (Ly6C^high^), intermediate (Ly6C^int^) and non-classical (Ly6C^low^) subsets as previously published (39) and evaluated as percentage of all monocytes (**Supplementary Figure 1**).

### 2.6 Statistical analysis

Samples were measured in duplicates and mean values were used for statistical analyses using GraphPad Prism 10 software. Graphs show individual data points representing one animal as well as range and quartiles. Gaussian distribution of data was evaluated by Shapiro-Wilk test and results were analyzed by two-way ANOVA with Geisser-Greenhouse correction, matching data of individual mice. Samples of individual mice were used for multiple stimuli (PBS, ADP, CRP-XL, or PAR4-AP) and for multiple anticoagulants, time-points and temperatures, but not for different blood draw techniques. Differences between treatments were evaluated by multiple comparisons for each agonist using uncorrected Fisher’s least significant difference test. Mixed model analysis was performed in case of missing values. P-values < 0.05 were considered statistically significant. Different p-values p < 0.05, p < 0.01, p < 0.001, and p < 0.0001 are indicated as *, **, ***, and ****, respectively.

## 3 Results

### 3.1 Blood collection technique exerts limited influence on PLA formation or platelet-mediated activation of innate leukocytes

**Figure 1:**
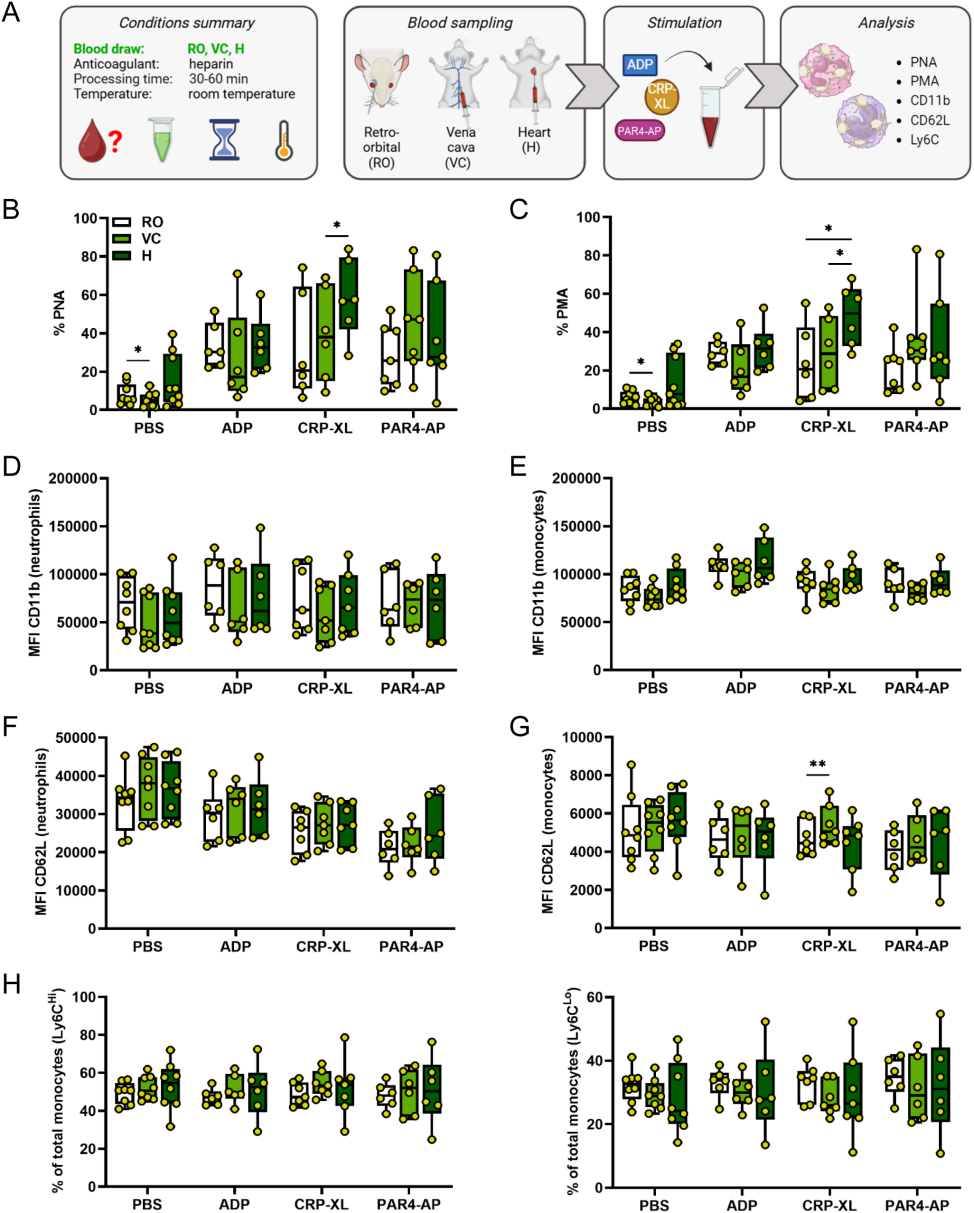
Blood collection technique exerts limited influence on platelet-leukocyte aggregate formation or platelet-mediated activation of innate leukocytes. **(A)** Experimental procedure: heparinized whole blood was collected from C57BL/6J mice using 3 different approaches: retro-orbital (RO), *vena cava* (VC), and heart (H) puncture. Samples were stimulated 30-60 minutes post-collection for 15 minutes at room temperature with phosphate-buffered saline (PBS) or with the following agonists: adenosine diphosphate (ADP; 250 μM), cross-linked collagen-related peptide (CRP-XL; 80 ng/ml), and protease-activated receptor 4-activating peptide (PAR4-AP; 50 μM). **(B)** Platelet-neutrophil aggregates (PNA) and **(C)** platelet-monocyte aggregates (PMA) were quantified. Neutrophil and monocyte activation was assessed by **(D, E)** CD11b expression and **(F, G)** CD62L shedding. **(H)** Monocyte polarization into (left) Ly6C^high^ classical and (right) Ly6C^low^ non-classical subsets was evaluated. n=6-9. *p<0.05 and **p<0.01. Panel A created in BioRender. https://BioRender.com/4kvfnq8.

First, we assessed the impact of blood sampling technique on PLA formation and platelet-mediated leukocyte activation using three different methods: retro-orbital sinus puncture (RO), *vena cava* puncture (VC), and heart puncture (H) (**Figure 1A**). Basal levels of PNAs and PMAs were significantly lower in VC- compared to RO-derived blood (PNAs mean ± SD: 5.23 ± 3.93% vs 7.93 ± 5.40%; PMAs mean ± SD: 3.57 ± 2.29% vs 5.57 ± 3.32%). Notably, while H-derived samples displayed high variability with a subset of samples showing markedly elevated basal PNAs and PMAs, overall levels were comparable to the other sampling techniques (**Figures 1B, C**). We found that the immunomodulatory response of platelets to specific agonists was only weakly influenced by the sampling method.

No significant differences in PLA formation were observed between the three blood collection techniques in response to ADP and PAR4-AP, but CRP-XL-induced PNA and PMA formation was increased in H-derived blood samples (**Figures 1B, C**). In contrast, CD11b expression and CD62L shedding of both neutrophils and monocytes as well as monocyte polarization were unaffected by blood sampling method irrespective of the agonist (**Figures 1D–H**). Of note, when sample stimulation was performed at 37°C instead of RT, differences in CRP-XL-induced PNA or PMA formation were no longer observable (**Supplementary Figures 2A–C**) and markers of innate leukocyte activation and polarization remained independent of blood draw technique (**Supplementary Figures 2D–H**).

Overall, these results demonstrate that blood sampling technique has minimal influence on platelet binding to innate leukocytes with augmented CRP-XL-induced PLA formation in H-derived blood, and no apparent effect on platelet-mediated leukocyte activation.

### 3.2 Calcium-chelating anticoagulants influence platelet-mediated leukocyte activation without affecting PLA formation

**Figure 2:**
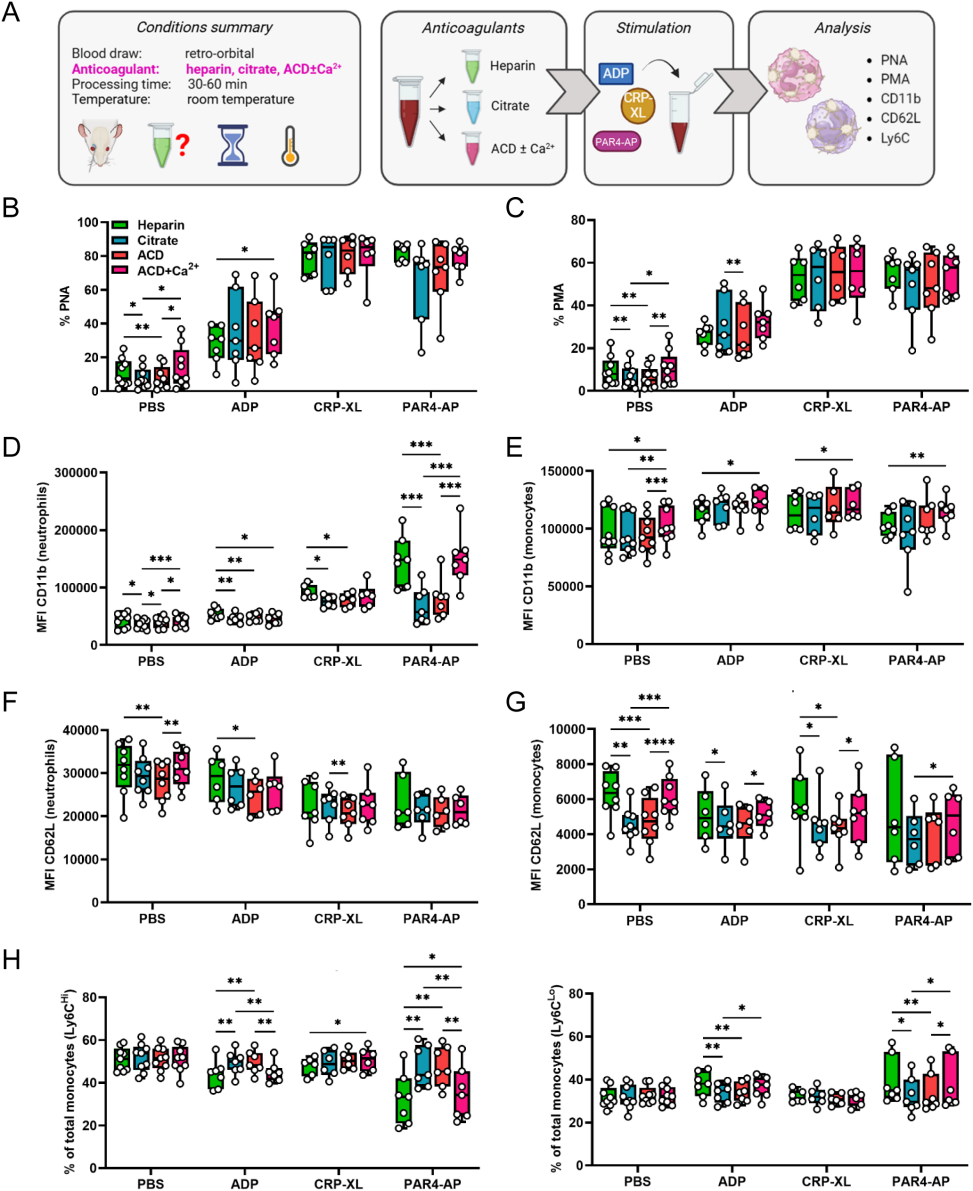
Calcium-chelating anticoagulants influence platelet-mediated leukocyte activation without affecting platelet-leukocyte aggregate formation. **(A)** Experimental procedure: whole blood was collected from C57BL/6J mice by retro-orbital puncture and anticoagulated with heparin, citrate, acid-citrate-dextrose (ACD), or ACD supplemented with 2 mM calcium (ACD+Ca^2+^). Samples were stimulated 30-60 minutes post-collection for 15 minutes at room temperature with phosphate-buffered saline (PBS) or with the following agonists: adenosine diphosphate (ADP; 250 μM), cross-linked collagen-related peptide (CRP-XL; 80 ng/ml) and protease-activated receptor 4-activating peptide (PAR4-AP; 70 μM). **(B)** Platelet-neutrophil aggregates (PNA) and **(C)** platelet-monocyte aggregates (PMA) were quantified. Neutrophil and monocyte activation was assessed by **(D, E)** CD11b expression and **(F, G)** CD62L shedding. **(H)** Monocyte polarization into (left) Ly6C^high^ classical and (right) Ly6C^low^ non-classical subsets was evaluated. n=6-9. *p<0.05, **p<0.01, ***p<0.001 and ****p<0.0001. **(A)** created in BioRender. https://BioRender.com/6p24kab.

Next, we investigated if platelet-leukocyte interactions are perturbed by the anticoagulant strategy adopted for blood sampling. As cellular analyses by flow cytometry require only small sample volumes, RO puncture with heparinized microhematocrit tubes is commonly used to obtain blood in murine models. Accordingly, we examined immunomodulatory platelet functions in heparinized blood with or without further addition of the calcium chelators citrate or ACD (**Figure 2A**), which lowered the blood pH to around 7.2 and 7.0, respectively (30). The effect of recalcification was also evaluated by supplementing ACD-anticoagulated blood with 2 mM CaCl_2_ (**Figure 2A**). Anticoagulation with citrate or ACD slightly lowered basal PNA and PMA levels relative to heparin alone, which were restored upon recalcification (**Figures 2B, C**). Again, we analyzed the effect of anticoagulation and recalcification in response to multiple agonists.

In general, calcium chelation with or without recalcification had negligible impact on agonist-induced PNA and PMA formation (**Figures 2B, C**), but influenced platelet-mediated leukocyte activation (**Figures 2D–H**). Interestingly, neutrophil and monocyte functions were differently affected by anticoagulation. While neutrophil CD11b expression was attenuated in the presence of calcium chelators in response to all agonists (**Figure 2D**), CD62L shedding was largely unaffected with only ACD samples showing minor changes (**Figure 2F**). Contrarily, calcium chelation had no effect on monocyte CD11b levels with recalcification leading to a slight upregulation (**Figure 2E**). However, citrate and ACD appeared to facilitate monocyte CD62L shedding while simultaneously preventing polarization, both of which were restored to heparin control levels upon recalcification (**Figures 2G, H**). Comparable results were obtained when samples were stimulated at 37°C, though effects of recalcification on PLA formation were more pronounced at these conditions (**Supplementary Figures 3A–H**).

Overall, calcium-chelating anticoagulants reduced baseline PNA and PMA levels, but did not significantly impact their formation following platelet stimulation. In neutrophils, calcium chelation impedes platelet-mediated CD11b upregulation, whereas monocytes display augmented CD62L shedding and impaired polarization. Of note, these effects were already observed in unstimulated blood, suggesting that calcium-chelation may also affect leukocyte activation independently of platelet-mediated fine-tuning.

### 3.3 Delayed sample processing modulates platelet-leukocyte interaction and fosters platelet-mediated leukocyte activation

**Figure 3:**
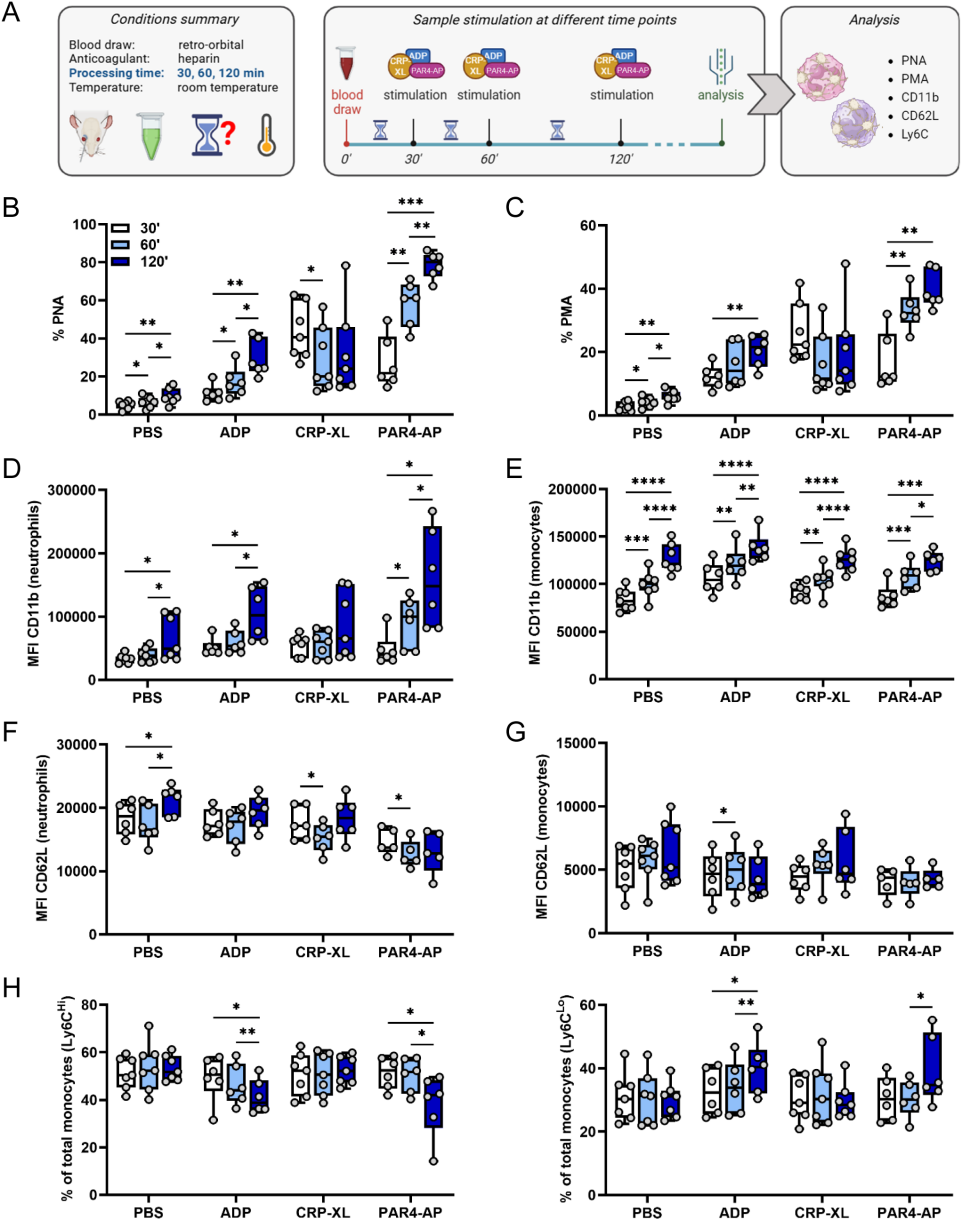
Delayed sample processing modulates platelet-leukocyte interaction and fosters platelet-mediated leukocyte activation. **(A)** Experimental procedure: whole blood was collected from C57BL/6J mice by retro-orbital sampling, anticoagulated with heparin and aliquots were stored at room temperature for 30, 60 or 120 minutes before stimulation with phosphate-buffered saline (PBS), adenosine diphosphate (ADP; 250 μM), cross-linked collagen-related peptide (CRP-XL; 80 ng/ml), and protease-activated receptor 4-activating peptide (PAR4-AP; 50 μM) for 15 minutes at room temperature. **(B)** Platelet-neutrophil aggregates (PNA) and **(C)** platelet-monocyte aggregates (PMA) were quantified. Neutrophil and monocyte activation was assessed by **(D, E)** CD11b expression and **(F, G)** CD62L shedding. **(H)** Monocyte polarization into (left) Ly6C^high^ classical and (right) Ly6C^low^ non-classical subsets was evaluated. n=6-7. *p<0.05, **p<0.01, ***p<0.001 and ****p<0.0001. **(A)** created in BioRender. https://BioRender.com/gcpv97i.

Infrastructural constraints of mouse facilities and the experimental need to sample multiple animals consecutively often necessitate delays in blood processing. During this time, storage at inherently non-physiological conditions might influence intercellular interaction and communication. To analyze if delayed processing affects platelet-mediated immunomodulation, whole blood was stored for 30, 60 and 120 min after sampling before stimulation with different agonists (**Figure 3A**). Basal levels of PNAs (**Figure 3B**) and PMAs (**Figure 3C**) were significantly elevated already after 60 min, and further increased at 120 min, displaying a ~2-fold increase over the observation period. This was accompanied by enhanced basal neutrophil and monocyte activation at 120 minutes as indicated by CD11b upregulation (**Figures 3D, E**). Of note, the agonist-induced PLA formation and leukocyte activation were differently modulated by delays.

A delay in sample processing time from 30 min to 60 min strongly augmented ADP- and PAR4-AP-induced PNA and PMA formation, but reduced PLAs induced by CRP-XL, though the latter didn’t reach statistical significance (**Figures 3B, C**). Additional delay of sample processing to 120 min after blood draw further exacerbated ADP and PAR4-AP hyper-responsiveness. Consistently, this increase in PLAs was accompanied by upregulation of CD11b over time (**Figures 3D, E**), while CD62L shedding was only marginally affected by the delay (**Figures 3F, G**). Further, delayed processing of 120 min fostered ADP- and PAR4-AP-induced monocyte polarization into non-classical Ly6C^low^ monocytes (**Figure 3H**). In contrast, delayed processing regulated neither CRP-XL-induced neutrophil activation nor monocyte CD62L shedding or polarization upon CRP-XL stimulation (**Figures 3D–H**).

Overall, delayed sample processing strongly increased platelet binding to innate leukocytes and facilitated platelet-mediated leukocyte activation and polarization induced by ADP and PAR4-AP, but CRP-XL responses were only slightly modulated. Of note, while CRP-XL-induced PLA formation declined over time, CD11b expression increased, suggesting that platelet-mediated fine-tuning may be overshadowed by direct delay effects on leukocytes.

### 3.4 Physiologic body temperature primarily affects PLA formation over platelet-mediated leukocyte activation

**Figure 4:**
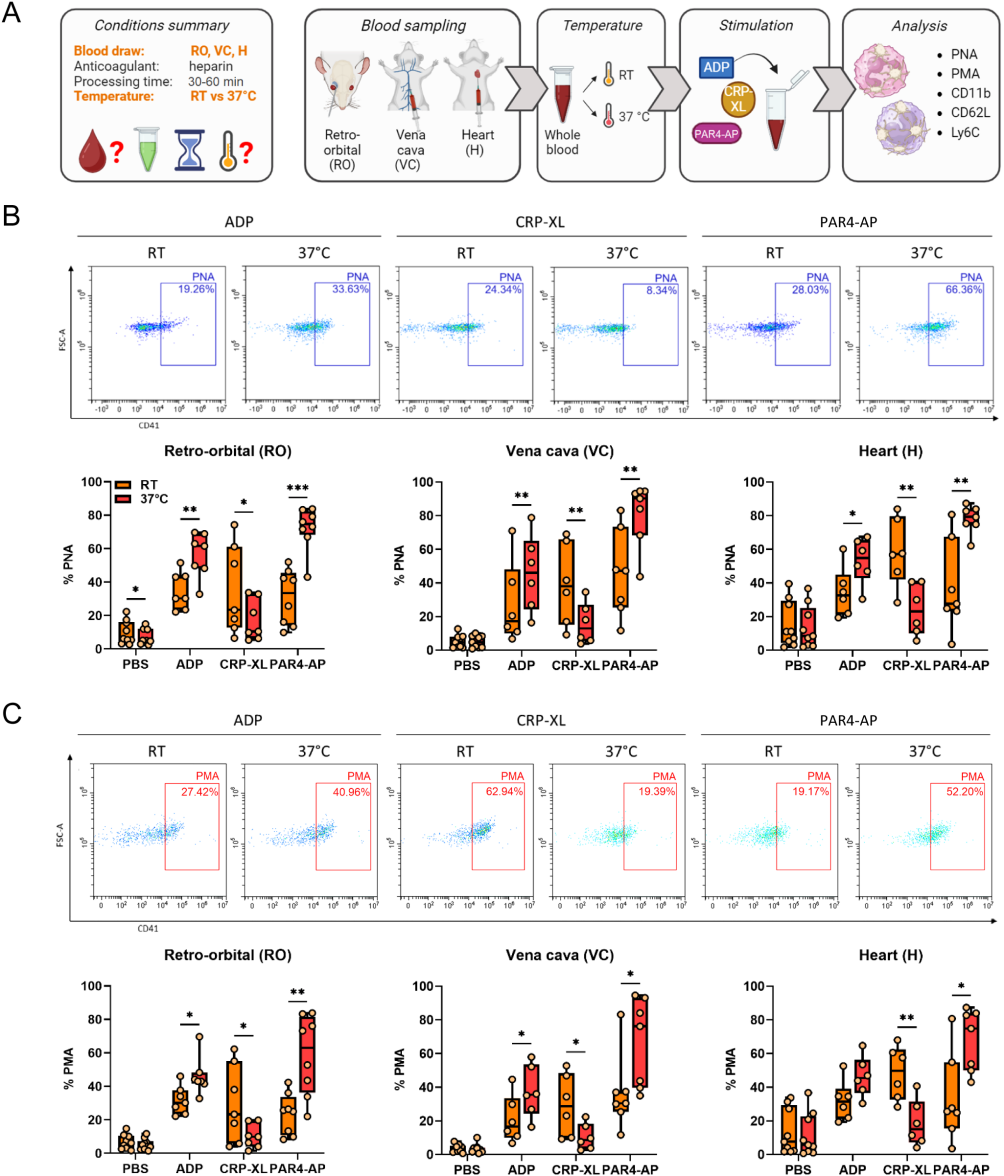
Experimental temperature modulates platelet-leukocyte aggregate formation regardless of the blood sampling technique. **(A)** Experimental procedure: heparinized whole blood was collected from C57BL/6J mice using 3 different approaches: retro-orbital (RO), *vena cava* (VC), and heart (H) puncture. Sample stimulation was performed 30-60 minutes post-collection at room temperature (RT) or 37°C. Samples were stimulated with phosphate-buffered saline (PBS) or with the following agonists: adenosine diphosphate (ADP; 250 μM), cross-linked collagen-related peptide (CRP-XL; 80 ng/ml), and protease-activated receptor 4-activating peptide (PAR4-AP; 50 μM). **(B)** Platelet-neutrophil aggregate (PNA) and **(C)** platelet-monocyte aggregate (PMA) formation was analyzed and representative dot plots of retro-orbital samples and quantifications are shown. n=6-9. *p<0.05, **p<0.01 and ***p<0.001. **(A)** created in BioRender. https://BioRender.com/6k3tup8.

**Figure 5:**
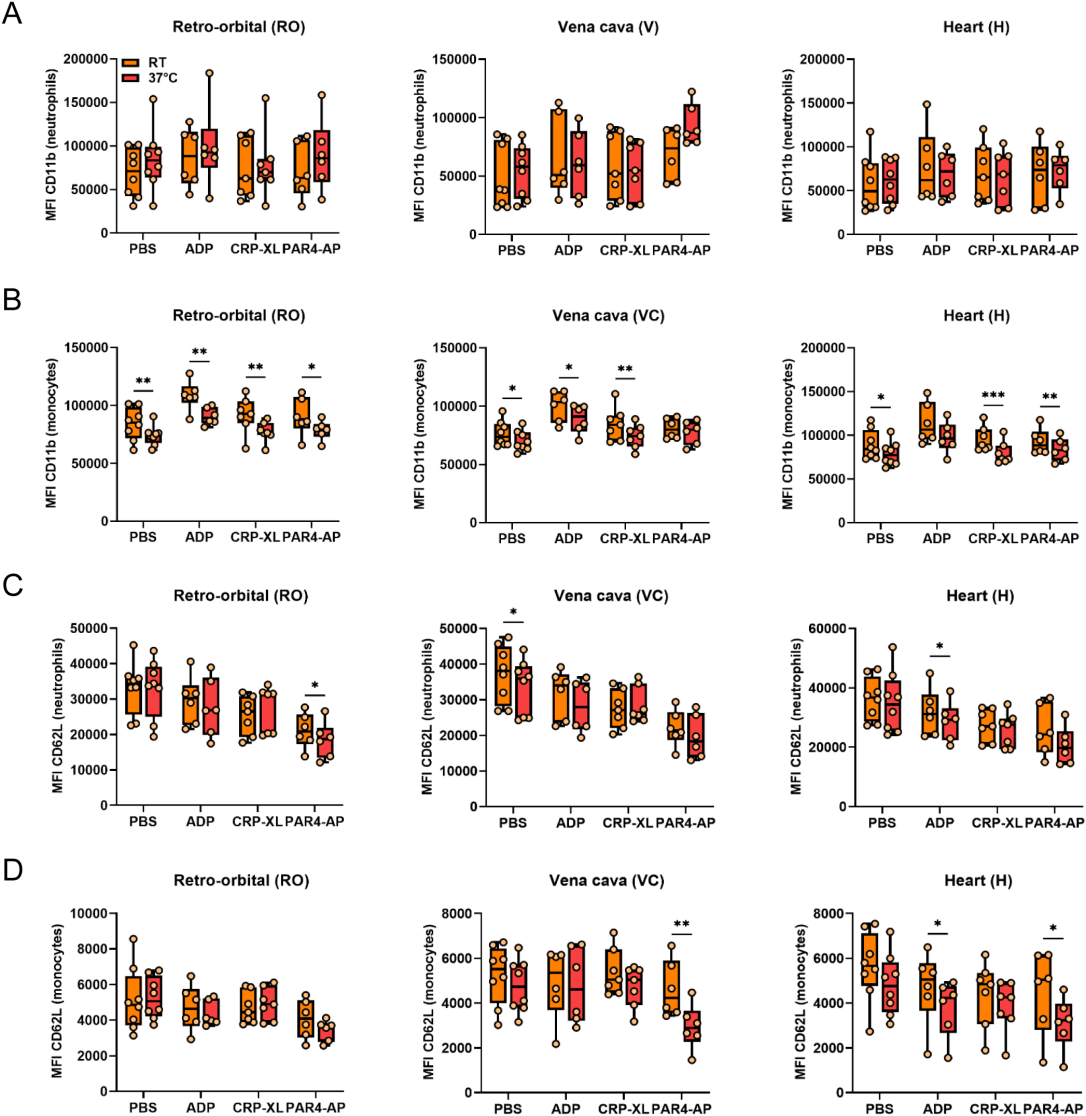
Experimental temperature weakly affects platelet-mediated neutrophil and monocyte activation independently of blood sampling technique. Heparinized whole blood was collected from C57BL/6J mice using 3 different approaches: retro-orbital (RO), *vena cava* (VC), and heart (H) puncture. Sample stimulation was performed 30-60 minutes post-collection at room temperature (RT) or 37°C. Samples were stimulated with phosphate-buffered saline (PBS) or with the following agonists: adenosine diphosphate (ADP; 250 μM), cross-linked collagen-related peptide (CRP-XL; 80 ng/ml), and protease-activated receptor 4-activating peptide (PAR4-AP; 50 μM). Neutrophil and monocyte activation was assessed by **(A, B)** CD11b expression and **(C, D)** CD62L shedding. n=6-8. *p<0.05 and **p<0.01.

The potential influence of incubation temperature on *in vitro* PLA formation and leukocyte activation and monocyte polarization was analyzed by stimulating whole blood obtained by different sampling methods (RO, VC, H) at RT or 37°C (**Figure 4A**). In general, the percentage of basal PNAs and PMAs was unaffected by incubation temperature, as only a slight difference was observed in RO-derived PNAs (**Figures 4B, C**). Interestingly, compared to RT, stimulation at 37°C augmented the ADP- and PAR4-AP-induced PLA formation regardless of the blood sampling technique, but PLA formation upon CRP-XL was significantly reduced (**Figures 4B, C**). However, this agonist-specific pattern of temperature-mediated changes in PLA formation was not reflected in leukocyte activation or polarization. On one side, CD11b expression by neutrophils was temperature-independent (**Figure 5A**), whereas monocyte CD11b was significantly reduced at 37°C (**Figure 5B**). On the other side, physiologic body temperature slightly fostered both CD62L shedding and monocyte polarization into non-classical monocytes at selected conditions (**Figures 5C, D**, **Supplementary Figure 4**).

We further adopted our protocol to investigate the potential interaction of temperature and anticoagulant (**Supplementary Figure 5A**). In general, the regulation of PLA formation (**Supplementary Figures 5B, C**) and leukocyte activation (**Supplementary Figures 6A–D)** by temperature was independent of the anticoagulant, though ADP-induced PLA formation and monocyte polarization were independent of temperature upon calcium chelation (**Supplementary Figures 5B, C**, **7**). Interestingly, in the presence of calcium chelators but not heparin alone, neutrophil CD11b followed the pattern of PNAs, pointing towards a role of direct interactions in these settings (**Supplementary Figures 5B**, **6A**). In contrast, temperature effects on monocyte CD11b and leukocyte CD62L were comparable between anticoagulants and uncoupled from PLA formation, with effects on CD62L being most pronounced in heparinized blood (**Supplementary Figures 6B–D**).

Collectively, these findings indicate that incubation temperature distinctly modulates PLA formation induced by ADP, CRP-XL, and PAR4-AP, independently of the blood sampling method or type of anticoagulant. Temperature effects on platelet-induced leukocyte activation are subtle and generally do not follow PLA patterns, with neutrophils and monocytes showing partially distinct regulation.

## 4 Discussion

This study investigated the impact of procedural variations such as blood sampling site, anticoagulant, processing delay, and incubation temperature, on murine platelet-leukocyte interactions. With growing recognition of the (patho-) physiologic roles of thromboinflammation and immunothrombosis, recent years have seen a rise in studies investigating platelet-leukocyte crosstalk across various clinical settings and murine models. Given this surge of interest and the methodological variability, a deeper understanding of how experimental factors influence PLA formation is essential to ensure reproducibility and to interpret potential discrepancies across studies.

We and others could previously show that human and murine platelets are highly sensitive to microenvironmental variations, which influence platelet activation and aggregation *in vitro*, including acidosis and alkalosis (24, 27, 40), hypo- and hyperthermia (25–27, 30), anticoagulation (23, 28, 30, 41), delayed processing (29, 30, 42), and platelet count (27, 43). However, whether immunomodulatory platelet functions are similarly susceptible to procedural changes remains poorly understood with only a handful of studies addressing the sensitivity of human PNA and PMA formation to alterations in sampling, temperature, platelet count, pH, anticoagulant, and storage time (27, 44–46).

In the current study, we extended these investigations to murine settings, demonstrating that agonist-induced PLA formation in murine blood was significantly influenced by processing delay and temperature, whereas blood sampling technique and anticoagulant had minimal influence on agonist responses. Protocol-induced changes in murine leukocyte activation and polarization did not consistently parallel PLA patterns, at least not within the early time points tested.

Our results emphasize that despite similarities in murine and human PLA sensitivity to some microenvironmental factors, species-specific differences underline the importance to confirm findings of murine models in human setting and *vice versa*. Moreover, the insensitivity of agonist-induced PNA and PMA formation to temperature in humans and to sampling site or anticoagulant in mice – variables with concomitantly profound impact on platelet function – suggests that PLA formation may represent a more robust marker for platelet activation than CD62P expression or GPIIb/IIIa activation in certain settings (27, 28, 30, 41, 47).

While murine platelet responsiveness differs between RO, VC and H blood (30), effects of sampling technique on PLA formation were limited to basal levels and CRP-XL sensitivity. These observations were somewhat unexpected, as murine blood sampling technique was previously shown to affect white blood cell but not platelet count (48) and platelet-to-leukocyte ratio influences agonist-induced PLA formation (27), suggesting that PLA levels may also differ by blood sampling. In line with our observations, previous studies in patient cohorts also reported only subtle and inconsistent differences in PLAs between arterial/venous or central/peripheral blood (44, 49, 50). In our setup, PLA regulation followed trends observed for platelet activation, suggesting that altered platelet responsiveness is the driving force for altered PLA formation. While we did not investigate the underlying mechanisms of how blood sampling influences murine platelet function or PLA formation, different tissue and blood chemistry and hemodynamic conditions may contribute to site-specific responses, e.g. due to variation in oxygenation and shear forces. Arterial and central venous blood also display slight differences in pH, which could influence CD62P-PSGL-1 binding (51, 52). In addition, extent and quality of tissue trauma during sampling may prime or pre-activate platelets to form PLAs, as RO sampling may injure capillary-adjacent tissue and lead to sample contamination with tissue fluid (53), while puncture of the tissue factor-rich myocardium could foster local thrombin generation (54).

Similarly, the influence of anticoagulant on murine PLA formation was minimal and limited to basal PLA levels, mirroring effects observed in human samples. In both species, calcium-chelating agents such as citrate, ACD or ethylenediaminetetraacetic acid (EDTA) yield lower basal PLA levels than thrombin inhibitors such as heparin, hirudin or phenylalanyl-prolyl-arginine chloromethyl ketone (PPACK) (45, 46, 55), reflecting the pivotal and species-independent role of calcium signaling for cellular functions including platelet degranulation as well as the calcium dependency of CD62P-PSGL-1 binding (56, 57). Again, PNAs and PMAs exhibited greater robustness than platelet CD62P or active GPIIb/IIIa against anticoagulation variation, which affects sensitivity of murine and human platelets to various agonists (30, 41). This anticoagulant-independent PNA and PMA formation upon platelet stimulation suggest that remaining extracellular calcium and/or dense granule-derived calcium released by activated platelets is sufficient to form PLAs. In line with previous findings of augmented monocyte and neutrophil responsiveness in heparinized compared to citrated blood (58), monocyte and neutrophil parameters also exhibited anticoagulant-associated variability in our setup. Interestingly, patterns were more reminiscent of platelet degranulation than PLA formation, pointing towards a putative role of platelet immunomodulation via soluble mediators in this experimental setting (30).

In contrast, delayed processing of murine blood samples profoundly impacted on basal and agonist-induced PLA formation as previously reported in human blood upon short-term storage (41, 50, 59). Whether the declining CRP-XL sensitivity associated with short-term processing delay we observed in murine blood also occurs in human setting currently remains unknown. However, prolonged storage of human platelets induces both spontaneous degranulation and GPVI shedding, thus increasing basal activation and dampening GPVI-mediated responses (60). Unphysiological storage conditions including stasis and oxidation may foster pro-coagulant and pro-thrombotic processes that facilitate low-key platelet activation and priming, e.g. via release of ADP or thromboxane A_2_. In addition, human platelets were shown to release matrix metalloproteinase (MMP) 1 and MMP2 during activation and PLA formation, which cleave PAR-1 at a cryptic ligand site, stimulating G_q_ and G_12/13_-induced signaling events and thereby priming platelets for full activation by other stimuli (61, 62). Although currently unexplored, murine platelets may employ a similar mechanism of protease-induced priming via PAR4 cleavage. Processing delay effects on PLA formation and innate leukocyte activation exacerbated over time, highlighting the importance of staggered analyses to prevent artifactual bias between experimental cohorts. In experimental settings where staggered analyses with fixed time-points between sampling and analyses are unfeasible, e.g. due to large cohorts or short time intervals, sampling mice at randomized order may help to avoid bias, but at the expense of increased within-treatment variation.

We detected similar agonist-specific regulation of murine PLA formation by temperature with increased ADP- and PAR4-AP-sensitivity but decreased CRP-XL sensitivity at 37°C compared to RT, closely mirroring the effect of temperature specifically on platelet CD62P expression, but not CD40L or active GPIIb/IIIa, underlining the central role of CD62P for platelet-leukocyte binding under these conditions (30). Similar to the effects of storage, platelet handling at RT may lead to priming and auto-activation. Indeed, activity of the ADP-degrading ectonucleotidase CD39 is reduced at RT relative to 37°C, enhancing ADP feedback at hypothermic conditions and leading to increased basal PLA formation in hypothermic mice (25). Temperature also determines the rate of GPVI shedding, which could explain the impaired CRP-XL sensitivity we observed at RT (63). Notably, the regulation of PLA formation by temperature in human blood remains inconclusive. We and others did not detect any differences in ADP or PAR1-AP-induced PMAs or PNAs between 37°C and mild (31-34°C) or severe hypothermia (RT) despite profound regulation of platelet activation (27, 47). While further studies are needed to confirm this, the observed discrepancies between human and murine settings may reflect the profound species-specific differences in thermoregulation. Since humans generally generate sufficient heat as byproduct of metabolism, thermoregulation is oriented towards heat dissipation rather than conservation, which might explain the described insensitivity of human PLAs to hypothermia (64). In contrast, heat loss is a constant risk to mice, which have to invest over 30% of their energy expenditure into maintaining body temperature at ambient temperature. In times of forced hypometabolism due to fasting, mice can enter a state of controlled hypothermia, leading to core temperature fluctuations of up to 10°C (64). Increased PLA formation upon hypothermia could thus support immune responses at conditions of extreme systemic stress. Furthermore, depending on ambient temperature and induction dose, mice commonly develop hypothermia, rather than fever, during severe inflammatory or infectious conditions under standard housing conditions (65, 66). Hence, awareness of how temperature affects thrombotic and immunomodulatory platelet functions is crucial to distinguish e.g. pathogen-specific from general microenvironmental effects.

Notably, while the use of whole blood in our study preserves physiologic blood composition and prevents potential artifacts upon cell isolation, experimental setups did not allow us to distinguish platelet-mediated modulation of leukocyte function from direct effects of protocol variables. Also, larger samples sizes may be required to detect subtle effects. Due to unclear leukocyte sensitivity to ADP and PAR4-AP, we also cannot exclude off-target leukocyte stimulation. Thus, future studies using isolated cells and additional agonists are warranted to disentangle individual cellular contributions. In conclusion, our findings shed light on how common procedural variations in preparing murine blood samples – sampling site, anticoagulant, processing delay, and assay temperature – exert distinct influences on platelet-leukocyte interactions *in vitro*, underlining the critical need for fastidious assay optimization to support data reproducibility and comparability.

We therefore provide several recommendations that should be considered for optimizing the *in vitro* analysis and comparability of murine platelet-leukocyte interactions:

- Anticoagulant and sampling site can be freely chosen based on the experimental needs.
- The time between sample collection and processing should be standardized. If that is not possible, the sampling order should be randomized to prevent bias when comparing different genotypes or treatments.
- Assays should be performed at consistent temperature conditions. If the samples are processed at RT, temperature should be controlled as best as possible, to avoid that daily shifts in ambient temperatures could greatly influence the variability of the results.
- In order to best preserve baseline PLA levels, blood from *vena cava* should be anticoagulated with calcium-chelating agents and processed within 30 minutes.
- Agonist concentrations should be titrated for individual experimental setups, particularly when changing processing delays or temperature.
- When investigating inflammatory/infectious disease models or other models affecting body temperature, samples of control and challenged animals should be allowed to adjust to equal temperature before analysis in order to filter noxa-specific effects.
- Details of sampling site, anticoagulant, processing delay, and assay temperature should be specified in in-house protocols and publications to facilitate data reproduction.

## Data Availability

The original contributions presented in the study are included in the article/**Supplementary Material**. Further inquiries can be directed to the corresponding authors.
