# Supplementary material for: Experimental conditions shape in vitro formation of murine platelet-leukocyte aggregates
Source: Front Immunol. 2025 Sep 2;16:1637038. doi: 10.3389/fimmu.2025.1637038 (PMC12439260; doi:10.3389/fimmu.2025.1637038)
Supplement: Supplementary file 1 [file DataSheet1.pdf]

SUPPLEMENTARY FIGURES

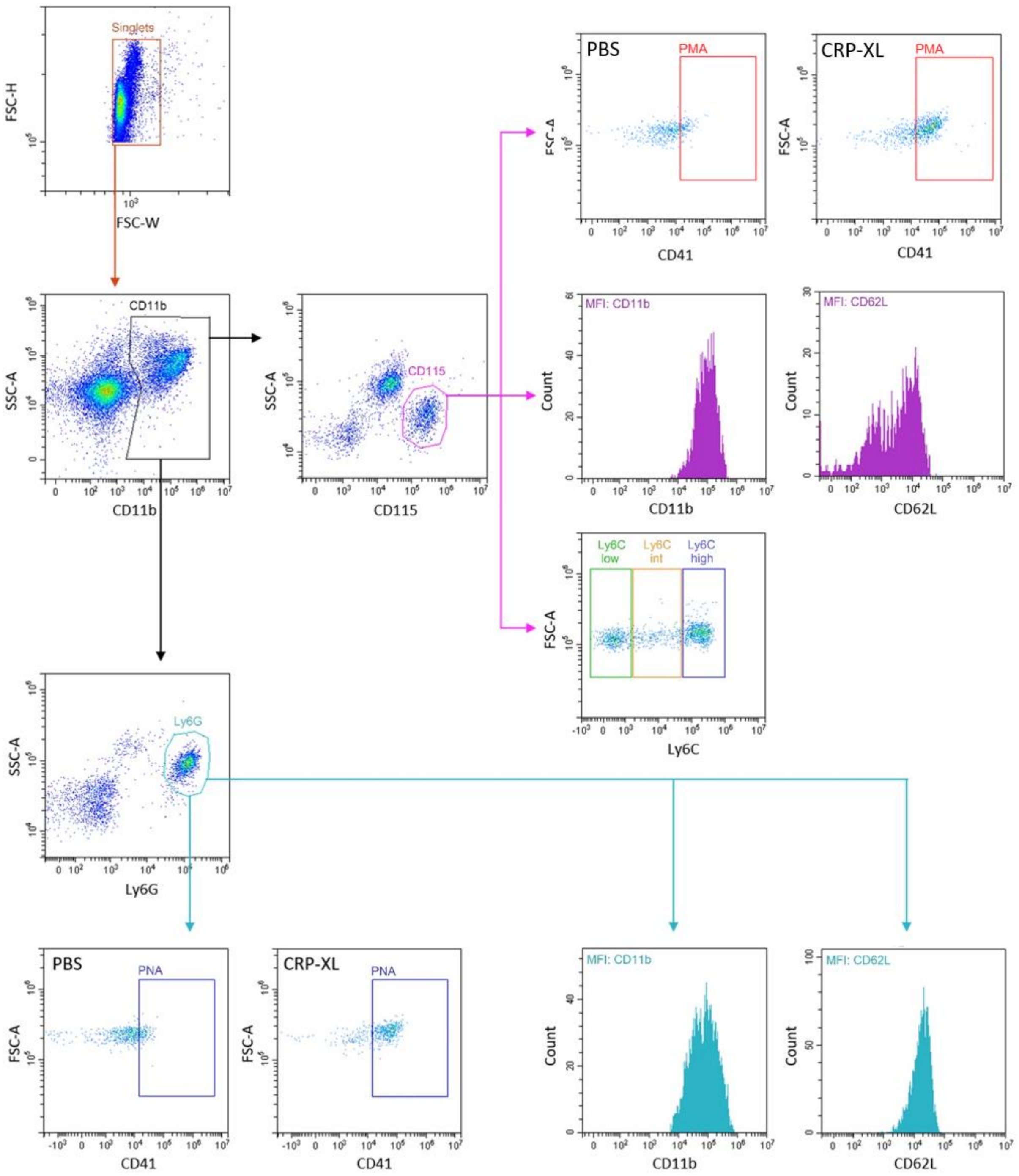

Supplementary Figure 1: Gating strategy to evaluate platelet-leukocyte aggregate formation and leukocyte activation. Single cells (singlets) were identified by comparing forward scatter height (FSC-H) and width (FSC-W). Myeloid leukocytes were identified as CD11b-positive and sub-categorized into CD115-positive monocytes and Ly6G-positive neutrophils. CD41-positive monocytes and neutrophils were identified as platelet-monocyte aggregates (PMA) and platelet-neutrophil aggregates (PNA). Activation of monocytes and neutrophils was further evaluated by CD11b expression and CD62L shedding. In addition, relative Ly6C expression was used to sub-divide monocytes into classical (Ly6C^{high}), intermediate (Ly6C^{intermediate}) and non-classical subsets (Ly6C^{low}), quantified as % of all monocytes. PMA, PNA and monocyte subsets were evaluated as % gated, CD11b and CD62L were quantified as mean fluorescence intensity (MFI). Plots show retro-orbital blood sample stimulated at room temperature CRP-XL, PMA and PNA plots also show control samples incubated with PBS.

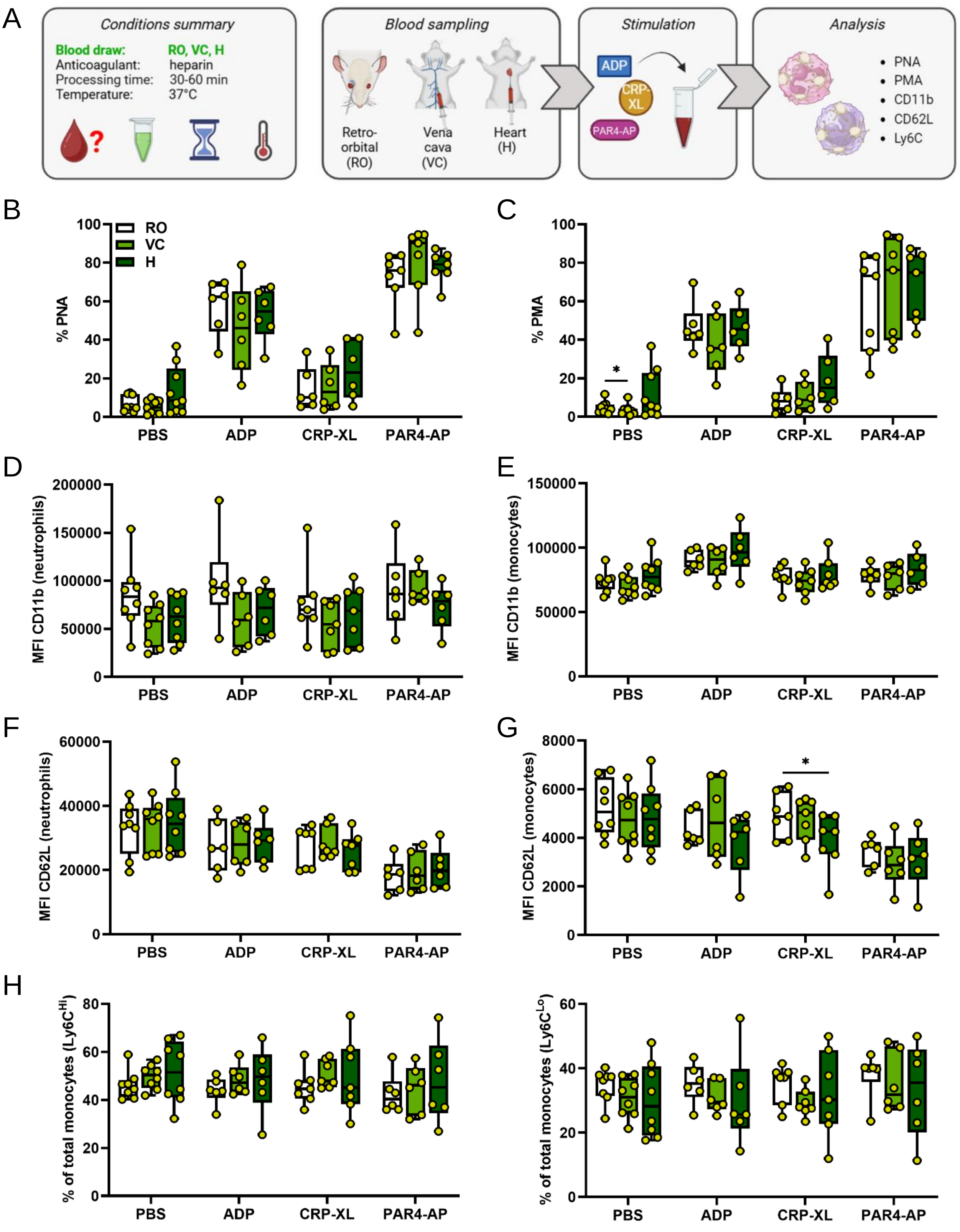

Supplementary Figure 2: Blood collection method does not affect platelet-leukocyte aggregate formation and platelet-mediated leukocyte activation at 37°C. (A) Whole blood was collected and processed as described in Figure 1. Sample stimulation was performed at 37°C. **(B)** Platelet-neutrophil aggregates (PNA) and **(C)** platelet-monocyte aggregates (PMA) were quantified. Neutrophil and monocyte activation was assessed by **(D-E)** CD11b expression and **(F-G)** CD62L shedding. **(H)** Monocyte polarization into (left) Ly6C^{high} classical and (right) Ly6C^{low} non-classical subsets was evaluated. n=6-9. *p<0.05. Panel A created in BioRender. https://BioRender.com/lxdbza2.

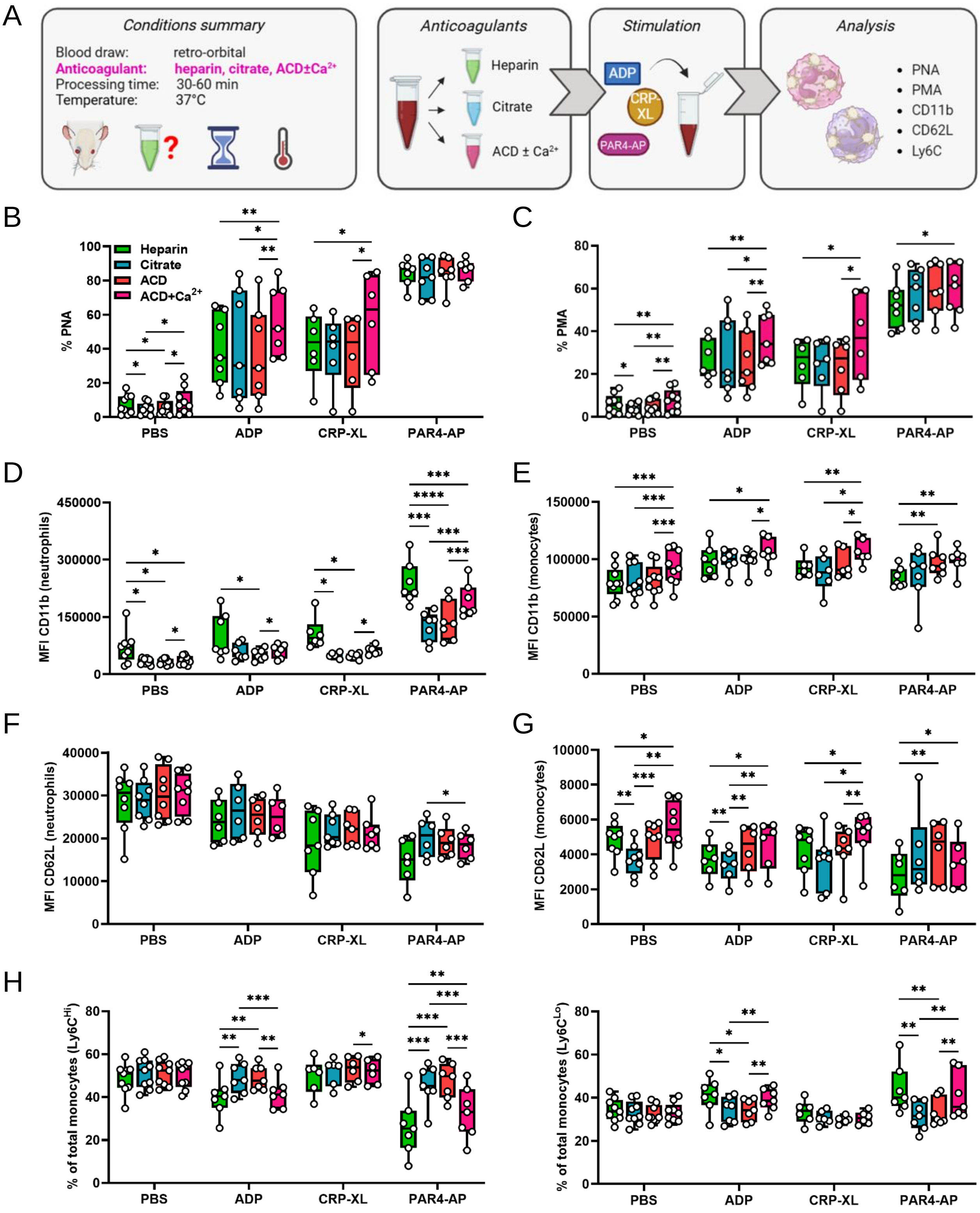

Supplementary Figure 3: Type of anticoagulant influences platelet-leukocyte aggregate formation and platelet-mediated leukocyte activation at 37°C. (A) Whole blood was collected and processed as described in Figure 2. Sample stimulation was performed at 37°C. **(B)** Platelet-neutrophil aggregates (PNA) and **(C)** platelet-monocyte aggregates (PMA) were quantified. Neutrophil and monocyte activation was assessed by **(D-E)** CD11b expression and **(F-G)** CD62L shedding. **(H)** Monocyte polarization into (left) Ly6C^{high} classical and (right) Ly6C^{low} non-classical subsets was evaluated. n=6-9. *p<0.05, **p<0.01, ***p<0.001 and ****p<0.0001. Panel A created in BioRender. https://BioRender.com/4f5xy2s.

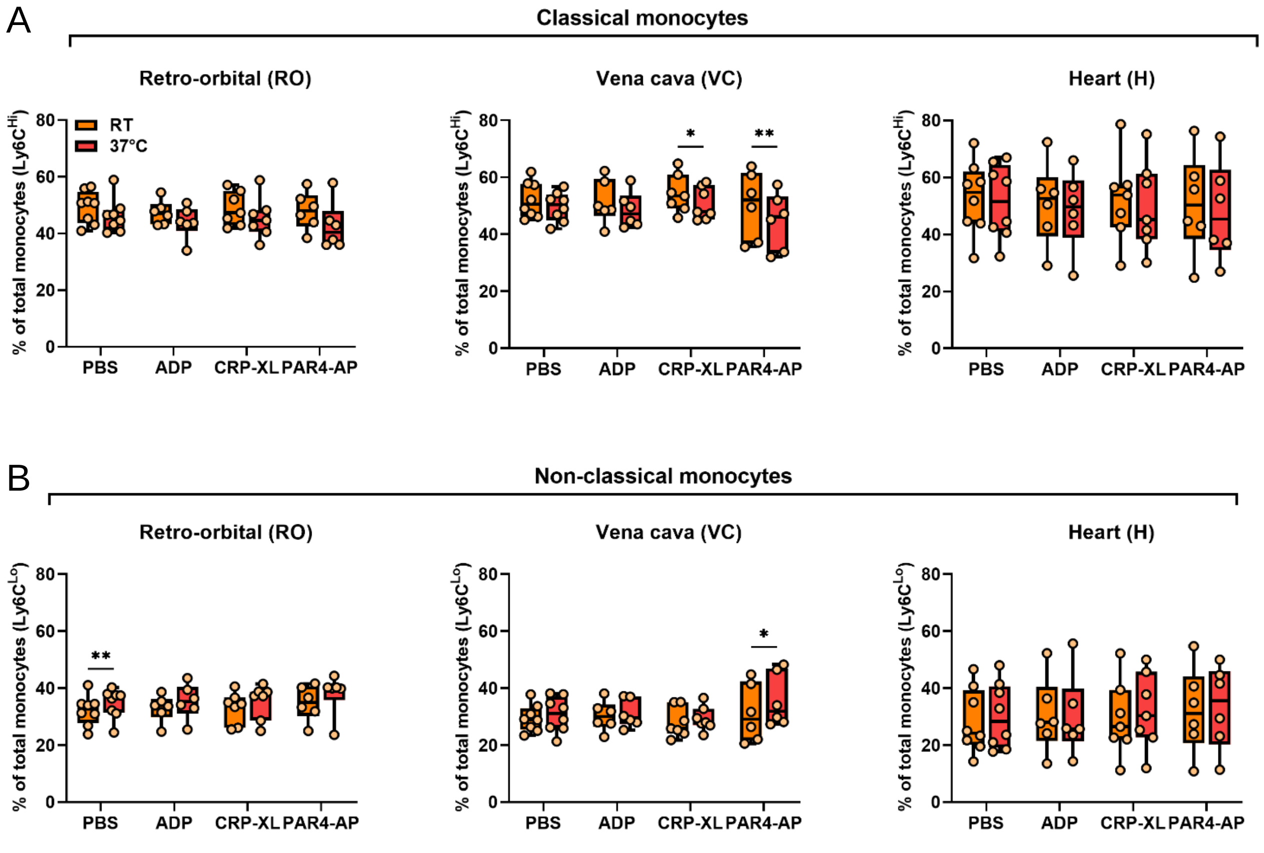

Supplementary Figure 4: Experimental temperature weakly influences monocyte polarization, independently of blood sampling conditions. Whole blood was collected and processed as described in Figure 4. Sample stimulation was performed at room temperature (RT) or 37°C. Monocyte polarization into **(A)** Ly6C^{high} classical and **(B)** Ly6C^{low} non-classical subsets was evaluated. n=6-8. *p<0.05, **p<0.01.

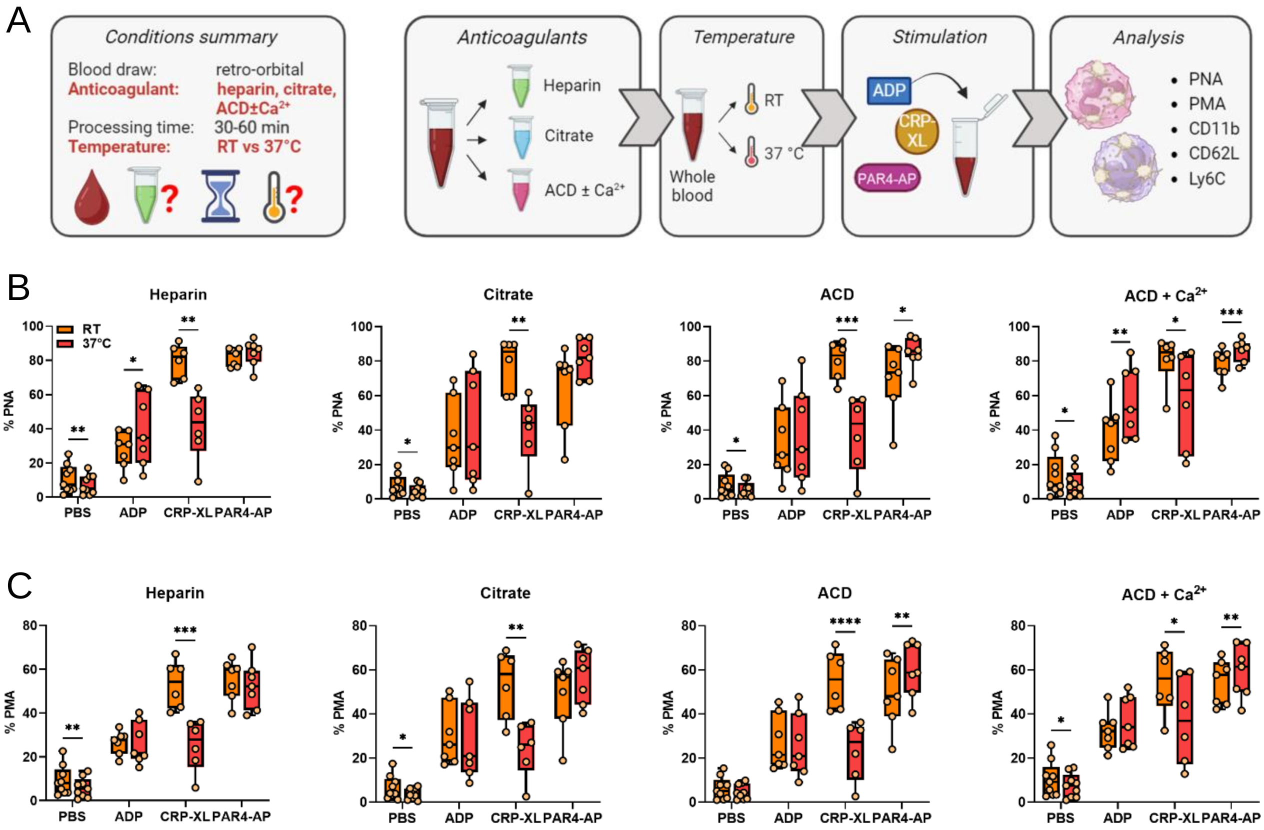

Supplementary Figure 5: Experimental temperature influences platelet-leukocyte aggregate formation independently of the anticoagulant. (A) Whole blood was collected and processed as described in Figure 2. Sample stimulation was performed at room temperature (RT) or 37°C. **(B)** Platelet-neutrophil aggregates (PNA) and **(C)** platelet-monocyte aggregates (PMA) were quantified by flow cytometry. n=6-9. *p<0.05, **p<0.01, ***p<0.001 and ****p<0.0001. Panel A created in BioRender. https://BioRender.com/4mbcmjt.

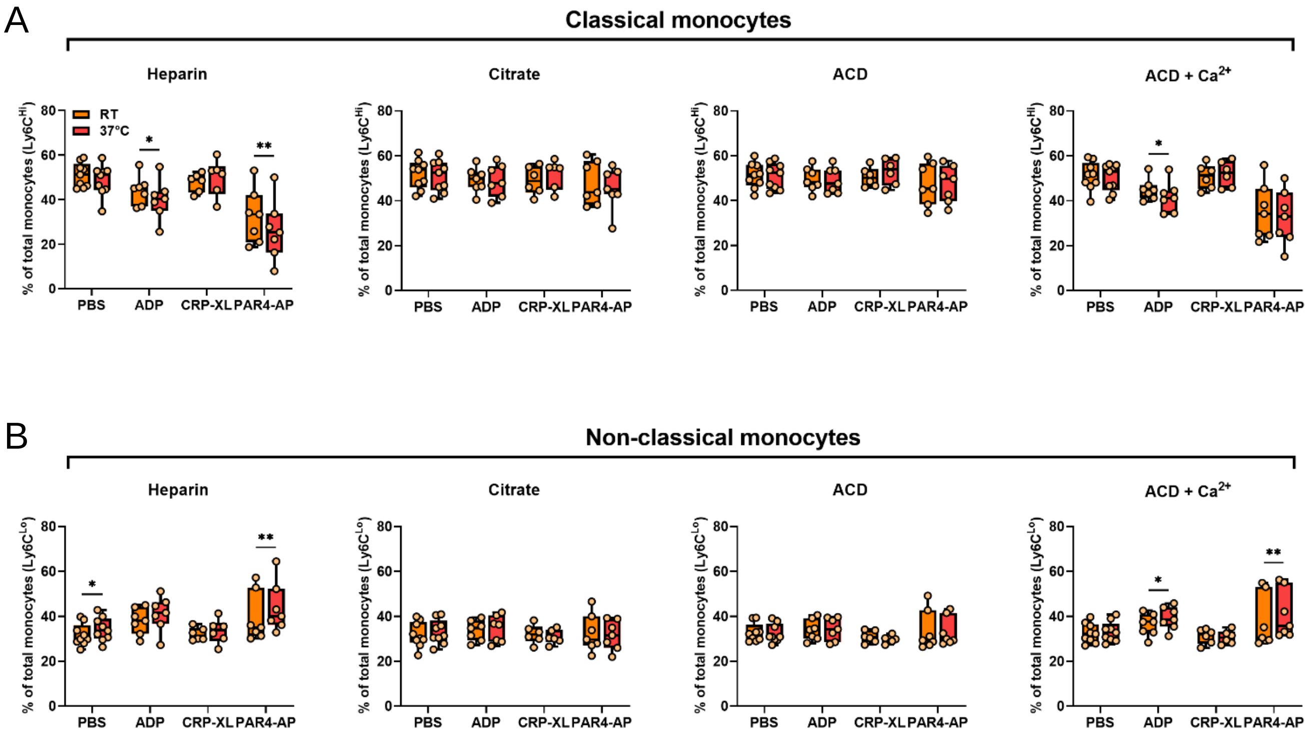

Supplementary Figure 6: Experimental temperature weakly influences monocyte polarization, independently of the anticoagulant. Whole blood was collected and processed as described in Figure 2. Sample stimulation was performed at room temperature (RT) or 37°C. Monocyte polarization into **(A)** Ly6C^{high} classical and **(B)** Ly6C^{low} non-classical subsets was evaluated. n=6-9. *p<0.05, **p<0.01.

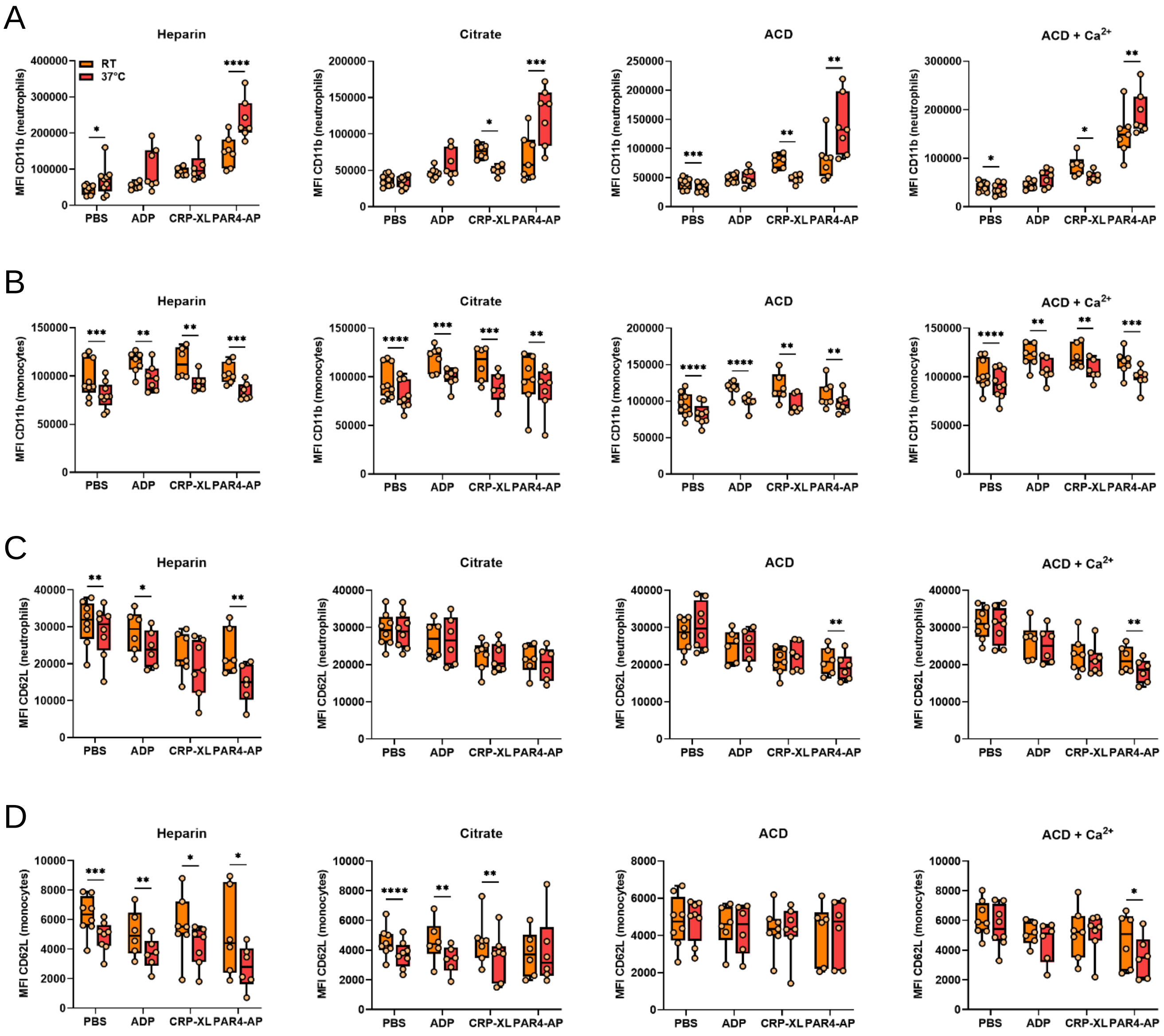

Supplementary Figure 7: Experimental temperature influences platelet-mediated leukocyte activation, independently of the anticoagulant. Whole blood was collected and processed as described in Figure 2. Sample stimulation was performed at room temperature (RT) or 37°C. Neutrophil and monocyte activation was assessed by **(A-B)** CD11b expression and **(C-D)** CD62L shedding. n=6-8. *p<0.05, **p<0.01, ***p<0.001 and ****p<0.0001.
